# Assays for predicting and monitoring responses to lung cancer immunotherapy

**DOI:** 10.7497/j.issn.2095-3941.2015.0019

**Published:** 2015-06

**Authors:** Cristina Teixidó, Niki Karachaliou, Maria González-Cao, Daniela Morales-Espinosa, Rafael Rosell

**Affiliations:** ^1^Pangaea Biotech, Quirón Dexeus University Hospital, Barcelona 08028, Spain; ^2^Dr. Rosell Oncology Institute, Quirón Dexeus University Hospital, Barcelona 08028, Spain; ^3^Cancer Biology and Precision Medicine Program, Catalan Institute of Oncology, Hospital Germans Trias i Pujol, Badalona 08916, Spain

**Keywords:** Immunotherapy, lung cancer, programmed cell death 1 (PD-1), PD-1 ligand (PD-L1), antibody

## Abstract

Immunotherapy has become a key strategy for cancer treatment, and two immune checkpoints, namely, programmed cell death 1 (PD-1) and its ligand (PD-L1), have recently emerged as important targets. The interaction blockade of PD-1 and PD-L1 demonstrated promising activity and antitumor efficacy in early phase clinical trials for advanced solid tumors such as non-small cell lung cancer (NSCLC). Many cell types in multiple tissues express PD-L1 as well as several tumor types, thereby suggesting that the ligand may play important roles in inhibiting immune responses throughout the body. Therefore, PD-L1 is a critical immunomodulating component within the lung microenvironment, but the correlation between PD-L1 expression and prognosis is controversial. More evidence is required to support the use of PD-L1 as a potential predictive biomarker. Clinical trials have measured PD-L1 in tumor tissues by immunohistochemistry (IHC) with different antibodies, but the assessment of PD-L1 is not yet standardized. Some commercial antibodies lack specificity and their reproducibility has not been fully evaluated. Further studies are required to clarify the optimal IHC assay as well as to predict and monitor the immune responses of the PD-1/PD-L1 pathway.

## Introduction

Lung cancer is among the most common cancers and the leading cause of cancer-related mortality worldwide^1^. Treatment options for lung cancer patients vary according to cell type, stage of disease, molecular profile, and functional status. Non-metastatic lung cancer is generally treated with curative intent using surgery, chemotherapy, targeted therapy, radiation therapy, or a combined modality approach^2^^-^^8^. However, the majority of the patients are diagnosed with extensive diseases and inoperable lesions^9^^,^^10^. Therefore, systemic therapy has become a mainstay for lung cancer management. Systemic treatments with chemotherapy have not improved patient prognosis in the last decade^4^^,^^5^^,^^8^, thereby emphasizing the need for new therapeutic strategies, such as immunotherapy, either as an adjunct to surgery and/or as a conventional form of cancer therapy^11^^-^^13^.

Lung cancer has long been considered poorly immunogenic because of the inactivity of different non-specific agents, such as Bacillus Calmette-Guerin^14^^,^^15^, interferon (IFN)-alpha^16^, and interleukin-2^17^, as well as specific antibodies, such as trastuzumab^18^^,^^19^. However, emerging preclinical and clinical data suggest the opposite, and immunotherapy is currently widely investigated as a treatment for lung cancer^20^^,^^21^.

Immune checkpoints, which are inhibitory signaling pathways that can down-modulate the immune system responses of T cells, are pivotal in peripheral tissues and for maintaining immune self-tolerance. Among the many molecularly defined checkpoint proteins^22^^,^^23^, one of the most studied in lung cancer clinical trials is programmed cell death 1 (PD-1) receptor, also known as CD279 (cluster of differentiation 279), and its ligand (PD-L1), also known as B7-H1 or CD274^13^^,^^24^. We review the current literature on the PD-1 and PD-L1 pathways, with emphasis on PD-L1 as a potential predictive biomarker of response to anti-PD-L1 antibodies.

## PD-1 and PD-L1 pathway

PD-1 is a type 1 transmembrane protein of the immunoglobulin superfamily^25^. In addition to its full length isoform, different splice variants of this protein (not all of which have been thoroughly studied) have been identified^26^. PD-1 plays an important role in limiting immune-mediated tissue destruction at sites with ongoing inflammation and/or infection. This immunoregulatory receptor is expressed on the surface of activated immune cell types, including T cells, B cells, natural killer (NK) cells, NKT cells, dendritic cells (DCs), and macrophages^27^, and is highly expressed on the surface of exhausted T cells. However, although nearly all exhausted cells express high levels of PD-1, not all cells expressing high levels of PD-1 are exhausted. Given that its blockade can restore the function of exhausted T cells^28^^,^^29^, PD-1 is considered a key immune checkpoint receptor that is expressed by activated T cells^30^.

PD-1 binds two B7 family ligands, namely, PD-L1 and PD-L2 (B7-DC or CD273)^31^^,^^32^. This interaction decreases the ability of activated T cells to produce an effective immune response and prevents the immune system from rejecting the tumor^33^. Among the ligands belonging to the B7 family, including PD-L1, PD-L2, B7-H3, and B7-H4, PD-L1 is the major membrane inhibitory ligand and the most studied in non-small cell lung cancer (NSCLC) clinical trials^34^. PD-L1 is expressed broadly in hematopoietic cells, including DCs, macrophages, mast cells, T cells, and B cells, and in non-hematopoietic cells, including endothelial, epithelial, and tumor cells^35^^,^^36^.

Cancer cells can activate PD-L1 expression through various oncogenic signaling pathways, such as phosphoinositide 3-kinase/protein kinase B (PI3K/PKB)^37^, extracellular-signal-regulated kinases/mitogen-activated protein kinase (Erk/MAPK)^38^, anaplastic lymphoma kinase/signal transducers and activators of transcription 3 (ALK/STAT3)^39^, Janus kinase (JAK)/STAT^40^, and myeloid differentiation primary response gene 88/tumor necrosis factor receptor associated factor 6 (MYD88/TRAF6)^41^ or in response to inflammatory cytokines that are produced by the infiltration of immune cells, such as IFNs^42^^,^^43^. Factors that influence PD-L1 expression may also depend on cell type. The receptor-ligand interaction PD-1/PD-L1 has been investigated as a target for cancer treatment in all of these situations.

## Potential role of PD-L1 as a predictive biomarker for immunotherapy

Antibodies that target either PD-1 or PD-L1 are being developed to block ligand-receptor interaction and to improve antitumor immune response by allowing T cells to attack the tumor. To date, these antibodies have demonstrated exciting clinical responses against many cancer types.

PD-L1 is expressed in several tumor types, such as melanoma, glioblastoma, and cancers in lung, kidney, head and neck, stomach, colon, pancreas, breast, cervix, cervical, and ovarian cancer. This protein has also been observed in hematologic malignancies, such as multiple myeloma, lymphoma, and various leukemia types^20^^,^^44^^-^^49^.

PD-L1-positive cancers may indicate immune active tumors that could be sensitive to anti-PD-1 and/or PD-L1 therapies because of their correlation with poor prognosis in many of these malignancies, including lung adenocarcinoma^50^^,^^51^. However, the prognostic role of PD-L1 remains unclear. Other studies have found that the expression of PD-L1 is correlated both with better prognosis and no prognostic significance, making it difficult for researchers to make definitive conclusions^43^^,^^52^. Such discrepancies may be explained by the current use of non-standardized immunohistochemistry (IHC) techniques for measuring PD-L1 levels in tissue.

PD-L1 has dynamic expression, and its evaluation by IHC is not well standardized. Previous studies have used a range of various antibodies, treatments, tumor types, and criteria to determine the positivity of samples. Therefore, a coherent definition of PD-L1 positivity must be established to facilitate further study of PD-L1 as a potential biomarker for the PD-1/PD-L1 pathway blockade. Given the intrinsic heterogeneity of PD-L1 expression in many tumors, the present results must be interpreted with caution. However, a biomarker of the response to a specific immunotherapy treatment is yet to be found.

In contrast, although testing the biopsied tumor tissue remains a recommended method for mutation analysis, challenges associated with serial tumor biopsy, particularly in NSCLC, have spurred the search for non-invasive blood-based assays that allow the frequent assessment of biomarkers as a part of routine clinical care^53^^,^^54^. Plasma and circulating tumor cells have also been proposed as alternative platforms for biomarker analysis^55^^,^^56^. A recent phase III clinical trial that compared high-dose chemotherapy with a rituximab regimen with standard rituximab, cyclophosphamide, doxorubicin, vincristine, and prednisone (R-CHOP) in aggressive diffuse large B-cell lymphoma showed that the plasma PD-L1 protein in blood was associated with poorer prognosis for patients who were randomized within the R-CHOP arm^57^. Therefore, plasma PD-L1 protein could provide a promising alternative for monitoring PD-L1 levels with agents blocking PD-1/PD-L1 interaction, such as in advanced lung cancer.

## PD-1 and PD-L1 in lung cancer

Immunotherapy has shown promising results in early NSCLC clinical trials involving PD-1 or PD-L1 antibodies^58^. These results have renewed the enthusiasm for immunotherapy as a treatment modality for lung cancer. Several drugs that target either the PD-L1 or PD-1 receptor are currently in preclinical and clinical development (**Table 1**). The first phase I trial with nivolumab [a human PD-1 blocking monoclonal antibody (mAb)] showed that PD-L1 expression in tumor cells could serve as a predictive biomarker to discriminate which patients would benefit from treatment. Only tumors expressing PD-L1 demonstrated an objective response rate (ORR). Reliable responses were observed in both non-squamous (ORR, 12%) and squamous histologies (ORR, 33%)^30^. Another phase I study with nivolumab (NCT00730639) showed that both PD-L1-positive and PD-L1-negative patients responded with an ORR of 44% and 17%, respectively, although numerically higher ORR, longer progression-free survival, and overall survival (OS) were observed in PD-L1 positive patients^62^. Nivolumab received U.S. Food and Drug Administration (FDA) approval in March 2015 and can be used for treating patients with advanced squamous NSCLC that progressed on or after platinum-based chemotherapy according to the CheckMate 017 phase III trial, which included squamous NSCLC patients regardless of their PD-L1 status. Median OS demonstrated superior performance for patients treated with nivolumab (9.2 months) compared with patients treated with docetaxel (6 months). Other studies involving nivolumab are ongoing, such as the phase III trial NCT01673867 comparing OS of nivolumab with docetaxel in subjects with non-squamous NSCLC after failure to prior platinum-based chemotherapy. Also ongoing are an open-labeled, randomized, phase III trials of nivolumab *vs*. investigator’s choice of chemotherapy (gemcitabine, cisplatin, carboplatin, paclitaxel, or pemetrexed) as first-line therapy for stage IV or recurrent PD-L1-positive NSCLC, and a phase I study of nivolumab in combination with gemcitabine/cisplatin, pemetrexed/cisplatin, carboplatin/paclitaxel, bevacizumab maintenance, erlotinib, ipilimumab, or as monotherapy in patients with stage IIIb/IV NSCLC.

**Table 1:** PD-L1 expression by immunohistochemistry in different studies

Reference	Tumor type	Drug	IHC Ab	Cell location	Cut-off (%)	*n* (PD-L1)	PD-L1+ pt (%)	ORR (%) PD-L1+	ORR (%) PD-L1-
Topalian *et al.*^30^	Solid	Nivolumab	5H1	Tumor cells (mb)	5	42	59.5	36	0
D'Incecco *et al.*^59^	Lung	Gefitinib/Erlotinib	58810	Tumor cells	5	98	53.1	61.2	34.8
Powles *et al.*^60^	Bladder	MPDL3280A	SP142	Tumor cells	5	205	10.73	28.6	25.9
				IC			26.8	43.3	11.1
Herbst *et al.*^61^	Lung	MPDL3280A	SP142	Tumor cells (mb and cyto)	5	53	24	33	22
				IC			26	46.1	18.2
Grosso *et al.*^62^	Melanoma	Nivolumab	28-8	Tumor cells (mb)	5	38	45	44	17
Brahmer *et al.*^58^	Solid	Nivolumab	5H1	Tumor cells (mb)	5	9	44.4	75	0
Garon *et al.*^63^	Lung	Pembrolizumab	22C3	Tumor cells (mb)	50	824	23.2	42.3	14.8
Konishi *et al*.^64^	Lung	–	MIH1	Tumor cells (mb and cyto)	1	52	27.2	–	–
Dong *et al.*^44^	Lung	–	5H1	Tumor cells (mb and cyto)	10	21	95	–	–
Hamanishi *et al.*^50^	Ovarian	–	27A2	Tumor cells	Moderate intensity	70	68.6	80.2	52.6
Taube *et al.*^65^	Solid	Nivolumab	5H1	Tumor cells (mb)	5	41	56	39	6
				IC			56	35	11

Ab, antibody; cyto, cytoplasm; IC, immune cells; mb, membrane; ORR, objective response rate; pt, patient.

As another anti-PD-1 mAb, pembrolizumab received FDA approval in October 2014 and can be used for treating epidermal growth factor receptor (EGFR) mutation-negative and ALK rearrangement-negative NSCLC that has progressed on or after platinum-based chemotherapy. Approval was granted based on the results of a phase I trial by Garon *et al*.^63^, which showed that pembrolizumab had antitumor activity and a tolerable toxicity profile for patients with advanced NSCLC. Moreover, PD-L1 positivity in at least 50% of tumor cells was correlated with improved efficacy of pembrolizumab (response rate of 45.2%). Current or former smokers had a response rate of 22.5%, while non-smokers had a response rate of 10.3%. Pembrolizumab for treating NSCLC is currently subjected to clinical trials, such as a phase I trial among advanced PD-L1-positive NSCLC patients (NCT02007070), a phase II/III study involving two doses of pembrolizumab *vs*. docetaxel for patients previously treated with PD-L1 positive NSCLC (NCT01905657), and combination studies with ipilimumab or chemotherapy for NSCLC patients (NCT02039674).

BMS-936559 and MPDL3280A are anti-PD-L1 mAbs. BMS-936559 showed modest activity (ORR of 6%-17%) among patients with advanced cancers, including NSCLC, in a phase I multicenter trial (NCT00729664). Objective response (a complete or partial response) was observed in 5 of 49 evaluable NSCLC patients^36^. In a phase I study with anti-PD-L1 MPDL3280A, multiple tumor type responses (as evaluated by Response Evaluation Criteria in Solid Tumors, version 1.1) were observed among patients with tumors expressing high levels of PD-L1, especially when PD-L1 was expressed by tumor-infiltrating lymphocytes (TILs). A 46% ORR was reported in the cohort of patients with the highest PD-L1 positivity, 17% with moderate PD-L1 positivity, 21% with low intensity, and 13% with PD-L1-negative tumors^61^. Results of the phase II trials in the first and second lines and phase III trials of MPDL3280A were compared with those obtained when docetaxel was used for patients with locally advanced or metastatic NSCLC who failed platinum therapy (NCT01846416, NCT01903993, and NCT02008228, respectively). NCT02013219 is another interesting trial with MPDL3280A that combines phase Ib with tarceva for the treatment of EGFR- and NSCLC-positive patients.

PD-L1 is up-regulated in cancer and is expressed in tumor cells in 40%-50% of NSCLCs independent of tumor histology^51^^,^^59^. PD-1 is expressed on the majority of the TILs, and the presence of high levels of PD-1 on cytotoxic T lymphocytes suggests a reduced production of various cytokines and a proliferation of T cells^64^. A recent study suggested that PD-1 and PD-L1 checkpoint inhibitors could be more effective for NSCLC patients whose tumors showed somatic EGFR mutations. PD-L1 positivity was significantly associated with the presence of EGFR mutations, and PD-L1-positive patients had higher sensitivity to EGFR inhibitors, a longer time to progression from therapy, and better OS compared with PD-1-negative patients^66^^,^^67^.

Several new immune-based treatments for small cell lung cancer (SCLC) are currently in clinical development. These treatments include the mAb-targeting Delta-like ligand 4 (DLL4) demcizumab (NCT01859741) and nivolumab with or without ipilimumab (a mAb antibody against CTLA-4) (NCT01928394)^68^^,^^69^.

## Available antibodies for IHC expression

Several companies have developed different primary antibodies for analyzing both PD-1 and PD-L1 proteins by IHC. Some studies suggest that tumor PD-L1 expression that is detected by IHC may predict clinical responses to anti-PD-1/PD-L1 therapy^36^^,^^65^. Therefore, PD-L1 expression has emerged as a potential predictive biomarker, but conflicting results have been obtained about the correlation between PD-L1 expression and effect on patient survival. Each company has developed PD-L1 detection techniques in isolation, thereby hampering the prospective validation of these tests and standardization for PD-L1 positive quantification. These contradicting results may be attributed to the lack of sensitivity and robustness of the antibodies that are used for detecting PD-L1 by IHC in clinical trials as well as the use of frozen versus formalin-fixed paraffin-embedded (FFPE) specimens^70^.

The similarities among the PD-L1 antibodies that are used in trials, as well as the staining localization, threshold for signal detection, and test conditions, need to be investigated to obtain a robust protocol. Gadiot *et al*.^71^ compared the performance of 15 anti-PD-L1 human antibodies that were used in IHC in FFPE melanoma cases. These antibodies included one mAb from eBioscience (San Diego, CA; MIH1), two mAbs from Otto Madjic (University of Vienna, Vienna, Austria; 5-496 and 2-272), one mAb from MBL International (Woburn, MA; 27A2), nine mAbs from Alan Korman (Medarex, Princeton, NJ; 16E11, 9A6, 16A4, 6H3, ETM-79, ETM-80, 1105, 25C8E8.F8 and 24B10.G6.D7), one mAb from L. Chen (Johns Hopkins University, Baltimore, MD; 5H1), and one polyclonal antibody from ProSci/Sigma (Poway, CA; 4059). The rabbit polyclonal antibody 4059 was the only one that did not result in background staining and blocked binding to PD-L1 by pre-incubation with a PD-L1 fusion protein and had the ability to stain FFPE.

The phase I study of Topalian *et al*.^30^ in 2012 was performed among 296 patients (including 122 NSCLC patients) previously treated with nivolumab and showed that PD-L1 positive tumors by IHC (performed using the 5H1 clone) had an ORR of 36%, whereas PD-L1 negative tumors did not achieve any ORR. Clones 5H1 and 28-8 were also compared; the staining of membranous PD-L1 was tested in FFPE tissue samples comprising tumor cells and tumor infiltrating immune cells from NSCLC, melanoma, and renal cell carcinoma. Clone 28-8 demonstrated a better detection (higher histoscores) than 5H1, although binding abilities of these clones to membrane PD-L1 were similar^72^.

A recently published patent describes antibodies with specific sequences that bind to human PD-L1 as well as reveals the benefit of detecting PD-L1 expression in FFPE human tissue samples by IHC (WO/2014/100079)^73^. The authors compared five commercially available PD-L1 human antibodies from eBioscience (San Diego, CA; MIH1), R&D Systems (Minneapolis, MN; AF-156), US biological (Salem, MA; 22 and 22E), and ProSci/ Sigma (4059) with two new mAbs from Merck (Whitehouse Station, NJ; 20C3 and 22C3) and found that none of these commercial antibodies had the required joint robustness, specificity, or sensitivity for using IHC in FFPE. However, the 22C3 and 20C3 antibodies were jointly robust, specific, and sensitive. The ability of the 22C3 antibody to detect a range of PD-L1 expression in different tumor types was also assessed by IHC in FFPE sections from different tumor types, including lung cancer^74^.

Roche/Genentech and Bristol–Myers Squibb have developed different companion assays for PD-L1 expression, each with its own experimental PD-L1 antibody. These antibodies include Spring Biosciences clone SP142 (MPDL3280A, Genentech, South San Francisco, CA), Spring Biosciences clone SP263 (MEDI4736, AstraZeneca, London), and Dako clone 28-8 (nivolumab, Bristol-Myers Squibb, New York, NY). To date, the properties and concordance between these IHC antibodies have not been reported, and only clone SP142 is commercially available. The characteristics of all antibodies that are discussed in this section are listed in **Table 2**.

**Table 2:** Anti-human-PD-L1 antibodies

Clone N (mAb)/Catalog N (pAb)	Provider	Host organism	Reference
MIH1	eBioscience, San Diego, CA	Mouse	^64^^,^^71^^,^^73^
5-496	O. Majdic, University of Vienna Medical School, Vienna	Mouse	^71^
2-272	O. Majdic, University of Vienna Medical School, Vienna	Mouse	^71^
27A2	MBL International, Wobum, MA	Mouse	^50^^,^^71^
16E11	Medarex, Princeton, NJ	Mouse	^71^
9A6	Medarex, Princeton, NJ	Mouse	^71^
16A4	Medarex, Princeton, NJ	Mouse	^71^
6H3	Medarex, Princeton, NJ	Mouse	^71^
ETM-79	Medarex, Princeton, NJ	Rabbit	^71^
ETM-80	Medarex, Princeton, NJ	Rabbit	^71^
1105	Medarex, Princeton, NJ	Human	^71^
25C8E8.F8	Medarex, Princeton, NJ	Human	^71^
24B10.G6.D7	Medarex, Princeton, NJ	Human	^71^
5H1	L. Chen, Johns Hopkins University, Baltimore, MD	Mouse	^30^^,^^71^^,^^72^
4059	ProSci/Sigma, Poway, CA	Rabbit	^71^
AF-156	R&D Systems, Minneapolis, MN;	Goat	^73^
22	US biological, Salem, MA	Rabbit	^73^
22E	US biological, Salem, MA	Mouse	^73^
20C3	Merck, Whitehouse Station, NJ	Mouse	^73^
22C3	Merck, Whitehouse Station, NJ	Mouse	^73^^,^^74^
SP142	Roche, Basel	Rabbit	^60^^,^^61^
SP263	Roche, Basel	Rabbit	^75^
58810	Abcam, Cambridge, UK	Rabbit	^59^
28-8	Bristol-Myers Squibb, New York, NY	Rabbit	^72^^,^^76^

mAb, monoclonal antibody; pAb, polyclonal antibody.

## Staining pattern and threshold for signal detection of PD-L1 protein expression

For an antibody to be considered as a favorable diagnostic tool, it must show sensitivity, specificity, reproducibility, and robustness in detecting the target by IHC. Some of the numerous available assays for detecting PD-L1 expression by IHC can only stain cancer cells, whereas the other assays can stain tumor-infiltrating immune cells. Therefore, patients with tumor cell staining and/or immune cell staining were examined in some studies, whereas the other studies only included patients with PD-L1 expression in tumor cells^30^^,^^60^^,^^74^. Given that these studies have not been compared, we could not ascertain whether the differences in these definitions can be attributed to antibody specificity, subjective interpretation, biological differences between the used immunotherapies, the nature of the analyzed patient tissues, or the technical issues that are related to tissue processing and storage. Taube *et al*.^43^ studied the predictive function of PD-L1 expression in cancer and immune cells, as well as that of PD-1 expression on the immune infiltrate. They found that PD-L1 expression in cancer and immune cells was highly associated with PD-1 expression in TILs, thereby indicating that PD-L1 expression reflects an immune reactive microenvironment.

Two patterns of cellular distribution of PD-L1, namely, membranous (cell surface) and cytoplasmic, have been described in tumor cells to indicate PD-L1 positivity. Membranous PD-L1 expression is present in tumors and inflammatory cells. PD-L1 staining pattern also differs between the assays. While some assays only evaluated membranous staining, others considered both membranous and cytoplasmic staining. PD-L1 is a type 1 transmembrane protein, and its cytoplasmic localization can represent intracellular stores of ligand that may relocate to the cell surface depending on cell stimulation. Interestingly, Brahmer *et al*.^36^ found that the membrane expression of PD-L1 was the most relevant biomarker for predicting the clinical response to PD-1 pathway blockade^65^. Moreover, the various PD-L1 protein expression staining patterns that are obtained in immune and tumor cells demonstrate that the scoring system used in clinical trials and the required percentages of positive cells in a positive sample can also vary.

The literature provides four definitions of PD-L1 sample positivity that are independent of the sample location and the staining of cells, i.e., whether ≥1, ≥5, ≥10, or ≥50 of cells per area are stained positive for PD-L1^44^^,^^58^^,^^61^^,^^63^. The specification of these parameters may explain why some patients who have been evaluated as PD-L1 positive respond to immunotherapy, whereas others do not respond. A standardized definition of PD-L1 positivity that links all the anti-PD-L1 antibodies by IHC must be provided to study the role of PD-L1 as a potential predictive biomarker for the therapeutic blockade of PD-1 and PD-L1. Without such definition, the comparison of clinical trial results using assays in different types of tumor will remain problematic.

## Conclusion

Immunotherapy for lung cancer is a new and exciting therapeutic modality. Multiple mAb candidates that target the PD-1/PD-L1 immune checkpoint have demonstrated reliable responses in tumors, including lung cancer, whereas some mAb candidates have shown remarkable antitumor effects in different clinical trials. Unfortunately, the lack of a reliable biomarker of response obscures such a scenario. Data on the correlation between PD-L1 positivity and patient responses to the different PD-1/PD-L1 blocking agents are also conflicting. PD-L1 is up-regulated in many cells and cancer types and contributes to the malignancy of these cancers by interacting with PD-1 and inhibiting T cell activation, thereby limiting the detection and destruction of tumor cells by the immune system. This ligand may play important roles in the inhibition of immune responses in both lymphoid and non-lymphoid organs. If PD-L1 is up-regulated in a tumor without an appropriate immune infiltrate, the blockade may have no effect because the tumor lacks the effector cells that fight the cancer.

Several immunohistochemical antibodies have been developed for detecting PD-L1 expression in FFPE tissue. Characterizing tumors and immune cells via PD-L1 protein expression by IHC may help identify those patients who can benefit from the mAb candidate anti-PD-1 and anti-PD-L1 agents. As a result, the ability of PD-L1 protein expression to become a favorable predictive marker of response has been measured in various ways in several clinical trials. The conflicting results of PD-L1 staining may be attributed to the small sample sizes that are tested in different assays and/or to the variability of the used antibodies. No precise cut-off has also been established for determining PD-L1 positivity by IHC. The limitations in the specificity and reproducibility of some antibodies may explain the contradictory relationships between assays.

Confirming PD-L1 as a predictive biomarker presents a promising new therapeutic opportunity to administer those agents that prevent PD-1/PD-L1 pathway interaction in advanced or metastatic lung cancers and other tumors. Further studies must be conducted to clarify the optimal IHC assay, validate and standardize the definition of PD-L1 positivity, and explore the relationship among various expression levels of the PD-L1 protein, as well as the effect of such levels on the prognosis of lung cancer patients with PD-1/PD-L1-directed therapies.
