# Erratum to Assays for predicting and monitoring responses to lung cancer immunotherapy

**DOI:** 10.7497/j.issn.2095-3941.2015.0055

**Published:** 2015-09

**Authors:** Cristina Teixidó, Niki Karachaliou, Maria González-Cao, Daniela Morales-Espinosa, Rafael Rosell

**Affiliations:** ^1^Pangaea Biotech, Quirón Dexeus University Hospital, Barcelona 08028, Spain; ^2^Dr. Rosell Oncology Institute, Quirón Dexeus University Hospital, Barcelona 08028, Spain; ^3^Cancer Biology and Precision Medicine Program, Catalan Institute of Oncology, Hospital Germans Trias i Pujol, Badalona 08916, Spain

In the published article^1^, one error appeared on page 89.

Pembrolizumab has not yet received FDA approval for NSCLC patients. In October 2014 pembrolizumab received FDA breakthrough therapy designation for lung cancer treatment supported by data from a phase Ib trial in previously treated NSCLC patients^2^.

The authors apologize for the errors and for any confusion it may have caused.
